# Frequent use of zinc-containing denture adhesive can cause neurological trouble: a case of severe copper deficiency myeloneuropathy visible in T2w-STIR sequences of spinal MRI

**DOI:** 10.1186/s12883-026-05280-y

**Published:** 2026-08-13

**Authors:** Isabel-Cathleen Helmsen, Martin Klietz, Julius Renne, Florian Wegner

**Affiliations:** 1 Department of Neurology, Hannover Medical School, Hannover, Germany; 2 Institute of Neuroradiology, Hannover Medical School, Hannover, Germany

**Keywords:** Copper deficiency myeloneuropathy, Zinc-containing denture adhesive, Spinal MRI, T2w-STIR sequences

## Abstract

**Background:**

We report a case of severe copper deficiency myeloneuropathy due to frequent application of zinc-containing denture adhesive. Our case report is supposed to enhance the clinical awareness for this preventable neurological disease.

**Case presentation:**

A 51-year-old patient presented with signs and symptoms of ataxia as well as spastic paraparesis and hyperreflexia. The patient reported hypesthesia with a sensory threshold at the level of T5 and a pallanesthesia of the lower extremities was determined. He could stand up with help but was unable to walk since May 2025.

Laboratory tests revealed markedly reduced serum levels of ceruloplasmin and copper whereas zinc was elevated. He had been using zinc-containing denture adhesive up to five times per day for his dental protheses since a trauma with multiple tooth extractions 25 years ago.

Standard MRI of the spine with T1- and T2-weighted MRI sequences was rated negative for any spinal cord abnormality. Only repeated MRI including T2w-STIR (short tau inversion recovery) sequences revealed a triangular signal enhancement in the cervical and upper thoracic spinal cord located in the posterior columns suggesting myelopathy.

Copper gluconate supplementation (2 mg/d) and omission of zinc-containing denture adhesive led to a normalization of serum copper levels and slow recovery of neurological deficits during the following weeks of rehabilitation. In 2026, the patient reported improved symptoms and was able to walk again with help for short distances.

**Conclusions:**

In cases of suspected hypocupremic myeloneuropathy and a negative standard MRI of the spine, additional T2w-STIR sequences should be used to provide morphological evidence for this diagnosis. Zinc-free denture adhesive should be recommended to prevent severe neurological conditions resulting from prolonged copper deficiency.

**Supplementary Information:**

The online version contains supplementary material available at 10.1186/s12883-026-05280-y.

## Background

Copper deficiency myeloneuropathy can be caused by a variety of medical conditions, including gastrointestinal diseases or following gastric surgery resulting in consecutive malabsorption and ceruloplasmin deficiency [1] as well as a side effect of different drugs, e. g. particular cancer treatment regimens leading to artificial serum copper deficiency [2]. Previously, one case of copper deficiency myelopathy caused by zinc-containing denture adhesive was reported showing subtle T2 hyperintensities within the dorsal column of the cervical spinal cord extending from C2 down to C7 level [3].

We present the case of a 51-year-old patient with hypocupremic myeloneuropathy resulting from long-term and frequent use of a zinc-containing denture adhesive. Neither myelopathy nor copper deficiency had been diagnosed for nearly one year after onset of sensory symptoms, progressive balance and gait disturbances in November 2024 which led to wheelchair-dependency in 2025. Spinal MRI revealed a triangular signal enhancement in the dorsal cervical and upper thoracic myelon along the posterior columns in the STIR sequence, following an initially unremarkable T2 sequence. Probably, this T2 negativity in routine spinal MRI was the main reason, why copper deficiency myelopathy in our patient had not been diagnosed during his stay in other hospitals in March and April 2025. However, it is important to emphasise that copper deficiency is in the differential diagnosis of MRI-negative myelopathy.

## Case presentation

The initial symptoms of the 51-year-old patient started in November 2024 as tingling paresthesia. At first, these sensory symptoms were limited to the distal upper extremities, over the course of a few weeks, they also became noticeable in the feet. Two months after initial symptoms a hypesthesia of the lower extremities evolved, ascending from distal to proximal. At the time of presentation in our hospital, this hypesthesia extended up to the level of T5. In addition, the patient described a deterioration in fine motor skills in both hands affecting daily activities. Regarding further motor involvement, the patient reported a weakness of the left arm, particularly in the distal region that had been present since initial manifestation. Two months after initial symptoms the patient first noticed unsteadiness of gait with a tendency to fall which progressed rapidly to spastically increased muscle tone, walking inability and wheelchair-dependency in May 2025. Recent infections/inflammations, cancer diseases, gastrointestinal surgery or spinal trauma were ruled out, however, he was involved in a street fight 25 years ago suffering jaw trauma with multiple tooth extractions. Relevant pre-existing conditions included intracranial stenosis in the M1 segment of the left middle cerebral artery (> 50% according to the Baumgartner criteria); the patient was taking 100 mg of aspirin and 40 mg of atorvastatin once daily. He was a current smoker (cumulative 40 pack-years); drug and alcohol use were denied. He was not on a specific diet and his nutrition was unremarkable. No neurological disorders were identified in the family history.

**Fig. 1:**
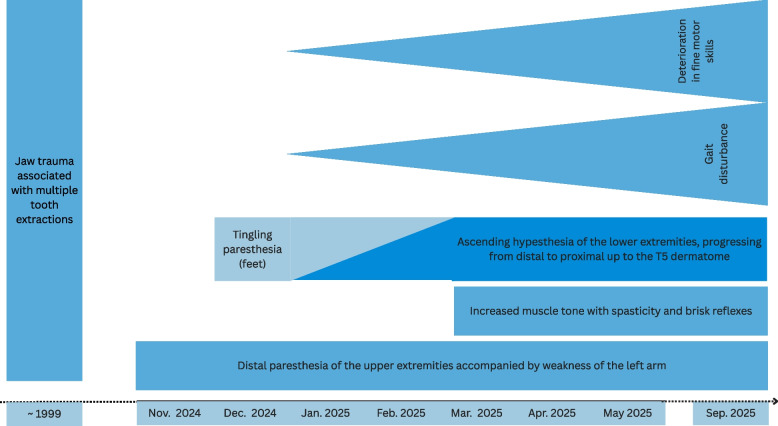
Chronological timeline of progressing copper deficiency myeloneuropathy symptoms due to long-term and frequent use of zinc-containing denture adhesive after jaw trauma

Ten months after symptom onset, the neurological examination in September 2025 revealed marked ataxia and spastic paraparesis with hyperreflexia in the lower extremities (see supplemental video file). Furthermore, the patient reported a sensory deficit from the legs up to the T5 level, and there was pallhypesthesia of the upper extremities (5/8) as well as loss of proprioception at the lower extremities. A chronological timeline of the evolving symptoms is shown in Fig. 1.

Laboratory tests revealed markedly reduced levels of ceruloplasmin (< 30 mg/L; normal range: 150–300 mg/L) and serum copper (0.8 µmol/L; normal range: 11.0–22.0 µmol/L). Relative exchangeable copper was within the normal range (12.5%). A decrease of copper was also observed in the 24-h urine collection (0.2 µmol/day; normal range: 0.4–1.1 µmol/day), which distinguishes the condition from Wilson’s disease [4]. In contrast, zinc was elevated in serum (20.5 µmol/l; normal range: 9–18 µmol/l). Regarding vitamin B12 metabolic status in serum, there was an elevation of homocysteine (20 µmol/L; normal range: < 12 µmol/L) and methylmalonic acid (87.2 µg/L; normal range: 9–32 µg/L) with normal levels for vitamin B12 (432 ng/L; normal range: 197–771 ng/L) and holotranscobalamin (68 pmol/L; normal range: 21–123 pmol/L), indicating a concomitant functional vitamin B12 deficiency. In addition, hypochromic anemia (Hb 11.8 g/dL; normal range: 13.5–17.2 g/dL; MCH 26.4 pg; normal range: 27.0–33.5 pg; MCV 82.1 fL; normal range 80.0–99.0 fL) with normal ferritin (75 µg/L; normal range: 27–365 µg/L) but reduced transferrin saturation (6%; normal range: 15–45%) and iron (4 µmol/L; normal range: 6–35 µmol/L) was detected. White blood cell counts did not show leukopenia (5.6 tsd/µL; normal range: 3.9–10.2 tsd/µL). There was no evidence of thrombocytopenia (264 tsd/µL; normal range: 150–370 tsd/µL). Antibodies associated with myelitis (anti-aquaporine-4, anti-MOG) or other autoimmune diseases all remained negative in serum. Cerebrospinal fluid analysis revealed a cell count of 3 cells/µl (normal range: 5 cells/µl) with regular protein levels of 0.44 g/l (normal range: 0.17–0.52 g/l) and slightly elevated lactate of 2.77 mmol/l (normal range: 1.2–2.1 mmol/l). The albumin quotient was increased at 7.25 indicating a mild blood–brain barrier dysfunction. Elevated neurofilament light chains in serum (112 pg/ml, > 2.5 z-score; normal: age-specific 95th percentile value 32 pg/ml) and cerebrospinal fluid (12,001 pg/ml, > 2.5 z-score; normal range: age-specific 95th percentile value at 1005 pg/ml) suggested a neurodegenerative process. Copper levels in the cerebrospinal fluid were also found to be markedly reduced (below 0.04 µmol/L; normal range: 0.12–0.37 µmol/L).

Electrophysiological testing revealed axonal polyneuropathy with a predominant involvement of the lower limbs. Somatosensory evoked potentials of the tibial and median nerves showed no reproducible cortical response. Motor evoked potentials were unremarkable.

**Fig. 2:**
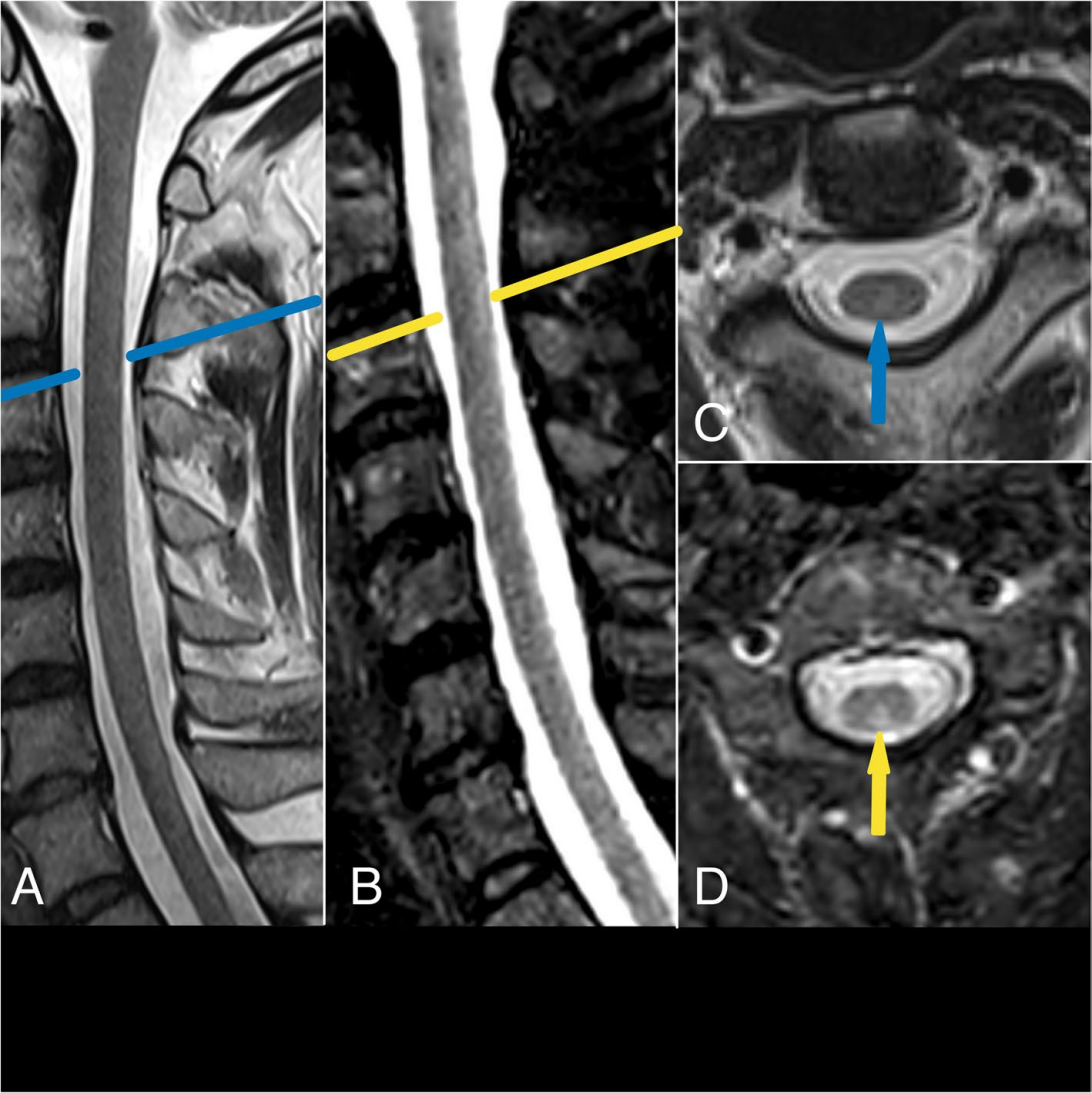
Spinal cord MRI of the patient with T2w-TSE (turbo spin echo) sequences with no apparent signal changes in (**A**) sagittal orientation and only questionable minimal signal changes in axial orientation (C, reconstruction plane indicated by blue line in A), rated as negative. In a repeated MRI, T2w-STIR sequences were added in sagittal (**B**) and axial orientation (D, reconstruction plane indicated by yellow line) with clear signal increase in the dorsal columns (yellow arrow) indicating myelopathy

Initial MRI of the cervical and thoracic spine including standard turbo spin echo T1w- and T2w-sequences as well as additional T1w-sequences after i. v. administration of gadolinium showed no signal changes in sagittal images (Fig. 2 A) and only very faint signal changes of the myelon on axial T2w-sequences (Fig. 2 C) that were non-continuous as well as in different locations and therefore rated as artificial. Cerebral MRI also remained negative for any causative pathology.

Overall, the findings suggested a disorder of copper metabolism. There were no indications of an alimentary, iatrogenic, or gastrointestinal origin of the symptoms. However, the patient had been using a zinc-containing denture adhesive up to five times per day on his protheses for approximately 25 years following trauma involving multiple tooth extractions. He regularly applied one tube per week with 47 g adhesive cream (33% calcium/zinc PVM/MA copolymer); if used properly, the content of the tube should have lasted for 5 to 6 weeks. He had switched adhesive cream types about 4 years ago, but previous formulations were likely also zinc-containing for better adhesion. Zinc is known to inhibit copper absorption by upregulating the enzymes in enterocytes that have a high affinity for copper, resulting in copper deposition and excretion [3, 5].

After routine spinal cord MRI of the patient with T2w-turbo spin echo sequences had shown only questionable minimal signal changes in axial orientation (rated as artificial), we wanted to use the T2w-STIR-sequence to detect myelopathy lesions with a higher sensitivity. Consequently, spinal MRI was repeated and T2w-STIR sequences were included. On this MRI a continuous T2w-signal increase following the dorsal columns from the cervical to the upper thoracic myelon was detected (Fig. 2  B and D). Thus, diagnostic findings were consistent with zinc-induced hypocupremic myeloneuropathy.

Copper gluconate supplementation (2 mg/d) and omission of zinc-containing denture adhesive led to a normalization of serum copper levels and slow recovery of neurological deficits during the following weeks of rehabilitation. Furthermore, tizanidine (12 mg per day), vitamin B12 supplementation (initially 1000 µg per day) and intensive physiotherapy were recommended. A combined (secondary role) of the vitamin B12 deficiency on the clinical course of the patient (e.g. incomplete recovery) cannot be excluded. In 2026, the patient reported improved sensory symptoms and was able to walk again using a walker for short distances.

## Discussion

Copper is an essential component of neuronal development, maturation, and function in the CNS [6]. Foods rich in copper include legumes, mushrooms, chocolate, nuts, seeds, and liver (all > 2.4 μg/g) [6]. Physiologically, copper is mainly absorbed in the upper small intestine primarily via the copper transport protein (CTR1) [6]. The liver serves as a storage organ; under the influence of the copper-binding protein ceruloplasmin, copper can be released into the plasma [5]. Following prior gastrointestinal surgery with subsequent malabsorption, a zinc excess is the second most common cause of copper deficiency, according to a systematic review of 55 case reports [4]. This is because zinc, which is also absorbed in the stomach and duodenum, induces the heavy metal-binding protein metallothionein [5]. Metallothionein binds copper within enterocytes facilitating its excretion in feces [5]. As clinical implication, the combination of any zinc-supplementation including denture adhesives and myeloneuropathy with hypochromic anemia in a patient should result in the appropriate serological tests to exclude copper deficiency.

Neurologically, copper deficiency can manifest as myelopathy, isolated peripheral neuropathy, motor neuron disease, myopathy, cerebral demyelination, cognitive dysfunction, and optic neuropathy [4]. There are also reports of cerebellar involvement [7]. On average, the onset of copper deficiency symptoms occurred after 22.2 years in patients without history of gastrointestinal surgery [4].

In addition, copper deficiency can manifest as hematological changes, including leukopenia and anemia [8]. Due to copper deficiency, ceruloplasmin oxidizes divalent to trivalent iron reducing the iron-binding capacity of transferrin, which could lead to iron-deficiency anemia [5], as seen in our case. We considered copper deficiency as principical cause of the patient’s neurological syndrome because all the copper-associated values and most hematological parameters indicated a severe copper deficiency including the typical hypochromic anemia. In relation to the hematological profile, vitamin B12 deficiency is typically associated with macrocytosis and hyperchromic anemia, whereas copper deficiency is characterized by hypochromic iron deficiency anemia [5] and neutropenia [8]. By assessing these hematological parameters and evaluating copper metabolism, a more precise differential diagnosis can be made to initiate the appropriate treatment in a timely manner.

According to a systematic review by Chen et al., which examined spinal MRI scans of 94 patients with hypocupremic myelopathy, cervical T2 hyperintensity was found in 60 cases (63.8%), typically appearing as a “V-shaped” pattern in the dorsal columns [9]. As initially in our case, an imaging correlate could not always be identified.

Due to the selective suppression of the MRI signal from fat containing structures by applying a selective excitation after a pre pulse with inversion of the spins, the sensitivity of detecting T2w-signal increases in the spinal cord is significantly higher in T2w-STIR sequences compared to standard turbo spin echo sequences, which was demonstrated e. g. for spinal cord lesions in multiple sclerosis [10]. By performing a supplementary spinal MRI including T2w-STIR sequences we were able to visualize the typical changes of hypocupremic myeloneuropathy in the form of a triangular signal enhancement in the dorsal cervical and upper thoracic myelon along the posterior columns. Therefore, we suggest that in cases of suspected hypocupremic myeloneuropathy and initially unremarkable or inconclusive spinal MRI, T2w-STIR sequences should be added to increase the sensitivity to diagnose this preventable neurological condition.

## Conclusions

This is the first reported case of zinc-induced copper deficiency myeloneuropathy detected by typical spinal cord signal changes only on a repeated MRI scan using T2w-STIR sequences following a negative standard MRI of the spine. Given that in a systematic review of 94 patients with hypocupremic myeloneuropathy only 60 (63.8%) showed cervical T2 hyperintensities [9], this should prompt the use of supplementary T2w-STIR sequences whenever hypocupremic myeloneuropathy is suspected. Generally, zinc-free denture adhesive should be recommended to prevent this severe neurological disease resulting from prolonged copper deficiency.

## Supplementary Information

Supplementary Material 1.

## Data Availability

The data supporting the findings of this case report are available from the corresponding author on reasonable request.
